# G-Quadruplex DNA and Other Non-Canonical B-Form DNA Motifs Influence Productive and Latent HIV-1 Integration and Reactivation Potential

**DOI:** 10.3390/v14112494

**Published:** 2022-11-11

**Authors:** Hannah O. Ajoge, Hinissan P. Kohio, Ermela Paparisto, Macon D. Coleman, Kemen Wong, Sean K. Tom, Katie L. Bain, Charles C. Berry, Eric J. Arts, Stephen D. Barr

**Affiliations:** 1 Schulich School of Medicine and Dentistry, Department of Microbiology and Immunology, Western University, Dental Sciences Building Room 3007, London, ON N6A 5C1, Canada; 2 Department of Family Medicine and Public Health, University of California San Diego, La Jolla, CA 92093, USA

**Keywords:** HIV, integration, latency, provirus reactivation, non-B DNA, guanine-quadruplex (G4) DNA, LEDGF/p75, CPSF6, reservoir

## Abstract

The integration of the HIV-1 genome into the host genome is an essential step in the life cycle of the virus and it plays a critical role in the expression, long-term persistence, and reactivation of HIV expression. To better understand the local genomic environment surrounding HIV-1 proviruses, we assessed the influence of non-canonical B-form DNA (non-B DNA) on the HIV-1 integration site selection. We showed that productively and latently infected cells exhibit different integration site biases towards non-B DNA motifs. We identified a correlation between the integration sites of the latent proviruses and non-B DNA features known to potently influence gene expression (e.g., cruciform, guanine-quadruplex (G4), triplex, and Z-DNA). The reactivation potential of latent proviruses with latency reversal agents also correlated with their proximity to specific non-B DNA motifs. The perturbation of G4 structures in vitro using G4 structure-destabilizing or -stabilizing ligands resulted in a significant reduction in integration within 100 base pairs of G4 motifs. The stabilization of G4 structures increased the integration within 300–500 base pairs from G4 motifs, increased integration near transcription start sites, and increased the proportion of latently infected cells. Moreover, we showed that host lens epithelium-derived growth factor (LEDGF)/p75 and cleavage and polyadenylation specificity factor 6 (CPSF6) influenced the distribution of integration sites near several non-B DNA motifs, especially G4 DNA. Our findings identify non-B DNA motifs as important factors that influence productive and latent HIV-1 integration and the reactivation potential of latent proviruses.

## 1. Introduction

An essential step in the life cycle of HIV-1 is the integration of its viral genome into the human genome. This event is permanent and leads to the life-long persistence of the virus within its host. Combination antiretroviral therapy (cART) suppresses productive HIV-1 replication in the infected individuals, thereby reducing the circulating virus to undetectable levels (reviewed in [1]). Despite this, resting memory CD4^+^ T-cells harbor an integrated virus that persists in a transcriptionally silent state, referred to as latency [2,3]. HIV-1 remains latent indefinitely until it is reactivated by means that are not fully understood, but include the cessation of antiretroviral therapy, the development of antiretroviral resistance, or clinically directed ‘shock and kill’ therapy [4,5,6]. Latency presents a major obstacle in curing an individual of HIV-1 infection. This is in part due to the slow decay rate of an individual’s latent reservoir after cART initiation, which has an estimated half-life of 44 months and an eradication timeline of >70 years in a patient [7,8]. Although the size of the latent pool is under much debate, modeling studies have suggested that the expansion and contraction of latently infected cells can generate low-level persistent viremia and intermittent viral blips that can replenish the latent reservoir [9,10,11]. As such, the characterization of the latent reservoir is essential for the eradication of the virus from the body.

Multiple mechanisms have been attributed to establishing and maintaining proviruses in a latent state and are likely not mutually exclusive. For example, the site and orientation of the integration, the availability of the cellular transcription factors and viral proteins, the epigenetic regulation of the HIV-1 promoter, and the microRNA regulation of chromatin remodeling and targeting of mRNAs have been shown to contribute to latency (reviewed in [12,13]). ‘Shock and kill’ strategies have been proposed to flush out latent HIV-1 reservoirs to effect a cure. The main objective of these strategies is to facilitate the reactivation of HIV-1 expression from latent reservoirs, which are then destroyed through either natural means (e.g., immune response and viral cytopathogenicity) or artificial means (e.g., drugs and antibodies) [14]. Many latency reversing agents have been used for reactivation, including physiological stimuli, chemical compounds (phorbol esters), histone deacetylase (HDAC) inhibitors, p-TEFb activators, and antibodies (e.g., anti-CD3); however, these agents fail to reactivate the entire pool of the latently infected cells, highlighting the importance of a single integration site in an infected cell [15,16,17,18]. Although somewhat controversial, several reports suggest that this failure is due to genomic location-driven differences in HIV-1 expression [5,16,18,19,20,21,22]. Specific integration sites are also associated with the clonal expansion of latently infected cells [23]. Clonally expanded cells have been shown to produce infectious HIV-1 in vivo, however, this may not be the case in all infected individuals [24,25]. In fact, the majority of chromosomally integrated HIV-1 proviruses are defective in infected individuals [26]. A better understanding of the molecular mechanisms contributing to the establishment and maintenance of latency will aid current eradication strategies.

Much of the early retroviral integration site analyses focused on the most frequent integration events to better understand the genomic environment surrounding sites that result in a productive infection. The cellular proteins lens epithelium-derived growth factor (LEDGF)/p75 and cleavage and polyadenylation specificity factor 6 (CPSF6) are important factors in determining HIV-1 integration site selection, particularly into transcriptionally active genes. Notably, CPSF6 directs the preintegration complex away from heterochromatin at the periphery of the nucleus and towards the gene-dense chromosomal regions, whereas LEDGF/p75 primarily functions to position the preintegration complex along gene bodies [27,28,29,30,31,32,33,34]. Comparatively, there are fewer integration site analyses with respect to latent infection. This is likely attributed to the rarity of latent integration events and the small number of cells comprising the latent reservoir. With improved sequencing technology and methods to isolate latently infected cells, the genomic environment surrounding latent proviruses has been recently studied in more detail to identify the common genomic predictors of a latent integration. For example, using five different in vitro models of latency, Sherrill-Mix and colleagues identified some genomic features (e.g., histone acetylation and alphoid repeats) which were associated with proviral expression in individual models [21]. Although they did not identify a common genomic predictor of latency across all of the models, the authors proposed that the features affecting latency were highly local and heterogeneous. Indeed, other groups have also shown that the latent reservoir is heterogeneous in nature [18,20,35]. The HIV-1 LTR promoter activity was also shown to be sensitive to the local chromatin environment and not controlled by DNA methylation or histone acetylation in cell lines [19,36]. Proviral insertion sites also affect the response to latency reversal agents (LRAs), where different LRAs can activate different subsets of proviruses in the latent population [18,20]. These different subsets are distinguishable in terms of the chromatin functional states and only represented <5% of cells carrying a latent provirus [18]. Recently, it was shown that individuals on prolonged antiretroviral therapy undergo selection of the intact proviruses with features of deep viral latency [37].

We previously identified non-B DNA motifs as a factor that potentially influences the HIV-1 integration site targeting [38]. Non-B DNA motifs are abundant in the human genome and form secondary structures using non-canonical Watson–Crick base pairing [39]. At least 10 non-B DNA conformations exist including A-phased repeats, inverted repeats, direct repeats, mirror repeats, short-tandem repeats, triplex repeats, G4, cruciform, slipped, and Z-DNA [39,40]. Several non-B DNA features preferentially act as the recipient of genetic information, stimulating homologous recombination >20-fold in human cells [41]. Some non-B DNA structures (e.g., G4, cruciform, triplex, and Z-DNA) have also been shown to potently silence the expression of adjacent genes [42,43,44,45,46,47,48,49,50,51,52]. In this study, we analyzed the HIV-1 integration site profiles of productively and latently infected cells and identified a correlation between non-B DNA motifs, provirus integration sites, and the reactivation potential of latent proviruses.

## 2. Materials and Methods

### 2.1. Cell Propagation, Virus Production and Infection

HEK293T cells (female) were obtained from the American Type Culture Collection and were maintained in standard Dulbecco’s Modified Eagle Medium (DMEM) or phenol red-free DMEM at 37 °C with 5% CO_2_ in a humidified incubator. All the media were supplemented with 10% heat-inactivated fetal bovine serum (FBS), 100 U/mL of penicillin, and 100 µg/mL of streptomycin. Pseudotyped HIV/VSV-G was generated by co-transfecting HEK293T cells with the plasmids p156RRLsinPPTCMVGFPWPRE (encoding the HIV vector segment), pCMVdeltaR9 (the packaging construct), and pMD.G (encoding the VSV-G envelope), as previously described [53]. The 293T cells, which were readily infected by HIV/VSV-G, were infected for 5 h with pseudotyped HIV-1/VSV-G in the presence of 10 µg/mL of polybrene (Sigma-Aldrich, St. Louis, MO, USA, #H9268-5G). The plasmids pHIV_GKO_ and pHIV_GKO-DU3LTR_ were kindly provided by Dr. Eric Verdin (Buck Institute). The pseudotyped HIV_GKO_ and HIV_GKO-DU3LTR_ viruses were similarly generated by co-transfecting HEK293T cells with pHIV_GKO_ or pHIV_GKO-DU3LTR_ and pMD.G for 48 h. Jurkat cells, which were readily infected by HIV_GKO_ and amenable to growth in suspension for 96 h, were infected via spinoculation at 3000 rpm at room temperature for 3 h in the presence of 10 µg/mL of polybrene (Sigma-Aldrich, #H9268-5G). Following the spinoculation, the virus was removed, and fresh medium was added to the well for 4 days.

### 2.2. Drug Treatment and Infection for Integration Site Analysis

BRACO-19 hydrochloride (BRACO-19) was purchased from Sigma-Aldrich (#SML0560-5MG). TMPyP4 was purchased from Calbiochem-EMD Millipore (#613560-25MG). 0.5 × 10^6^ HEK293T cells were plated in 6-well plates and left untreated (control) or treated for 24 h with either BRACO-19 (0, 1, 3, and 32 µM) or TMPyP4 (0, 0.5, 1, and 8 µM). The BRACO-19 and TMPyP4 concentrations were established from previously used concentrations [54,55,56]. The cells were then infected for 5 h with pseudotyped HIV-1/VSV-G in the presence of 10 µg/mL of polybrene (Sigma-Aldrich, #H9268-5G). The medium was changed 5 h post-infection and the cells were treated anew with either BRACO-19 or TMPyP4 for 24 h. The genomic DNA was extracted from the cells as per the manufacturer’s instructions using the DNeasy Blood and Tissue Kit (Qiagen, Hilden, Germany, #69504) and processed for the integration site profile analyses.

### 2.3. HIV Integration Site Library and Computational Analysis

Integration sites were determined from the sequence junction of the LTR and human genome sequences. The genomic DNA was processed for integration site analysis and sequenced using the Illumina MiSeq (San Diego, CA, USA) platform, as previously described [38,57]. Fastq sequencing reads were quality trimmed and the unique integration sites were identified using our in-house bioinformatics pipeline, which was described previously and is now called the Barr Lab Integration Site Identification Pipeline (BLISIP version 2.9) [38]. BLISIP version 2.9 includes the following updates: bedtools (v2.25.0), bioawk (awk version 20110810), bowtie2 (version 2.3.4.1), and restrSiteUtils (v1.2.9). The HIV-1 LTR-containing fastq sequences were identified and filtered by allowing up to a maximum of five mismatches with the reference NL4-3 LTR sequence. The LTR sequences matching any region of the human genome (GRCh37/hg19) were discarded. Sites located in various common genomic features and non-B DNA motifs were quantified. The heatmaps were generated using our in-house python program BLISIP Heatmap (BLISIPHA v1.0), which calculates the fold enrichment of sites in the desired distance bin from each feature compared to that of the desired control dataset. The matched random control datasets were generated from the hg19 reference genome to account for restriction site bias in the preparation of the libraries that used restriction enzymes in their construction, as previously described [38,58]. The random control datasets were generated from the hg19 reference genome using the random tool of BEDTools v2.28 for the libraries generated by random fragmentation (e.g., shearing) [59]. The sites that could not be unambiguously mapped to a single region in the genome were excluded from the study. All non-B DNA motifs were defined according to the previously established criteria [60]. The G-quadruplex algorithm identifies four or more individual G-runs of at least three nucleotides in length. The algorithm requires at least one nucleotide between each run and considers up to seven nucleotides as the spacer, including the guanines [60].

### 2.4. MTT Assay

HEK293T or Jurkat cells were treated with increasing concentrations of BRACO-19 (0, 1, 3, and 32 µM) or TMPyP4 (0, 0.5, 1, and 8 µM) for 5 days at 37 °C with 5% CO_2_. The cell metabolic activity was measured using the MTT (3-(4,5-Dimethylthiazol-2-yl)-2,5-Diphenyltetrazolium Bromide) kit (Thermo Fisher Scientific, Waltham, MA USA #M6494) according to the manufacturer’s instructions. The absorbance of the plates was read at 540 nm using the Agilent Biotek Epoch microplate spectrophotometer (Agilent Technologies, Santa Clara, CA, USA) plate reader and the Gen5 version 2.06 analysis software. The percent viability was calculated relative to the untreated control cells.

### 2.5. Confocal Immunofluorescence Microscopy

Jurkat cells were treated with 0, 8, or 32 µM of BRACO-19 for 24 h. Intracellular G4 structure antibody staining protocols were adapted from previously published protocols [61,62]. The cells were then fixed with 4% paraformaldehyde (Electron Microscopy Science, Hatfield, PA, USA) for 10 min and permeabilized with 0.5% Tween 20 in 1× PBS. The blocking was performed overnight at 4 °C with 5% goat serum (Wisent Bio Products, St-Bruno, Quebec, Canada) in 1× PBS. The cells were incubated for 24 h with an anti-G4(1H6) antibody (Millipore Sigma-Aldrich, Oakville, Ontario, Canada) at a ratio of 1:100. The cells were washed 5 times with 0.1% Tween 20 in 1× PBS (PBST). Alexa Fluor 594 (Thermo Fisher Scientific Life Technologies, Wyman Street, Waltham, MA USA) secondary antibody staining was performed at a ratio of 1:1000 for 2 h at room temperature. The cells were washed 3 times with PBST and 2 times with 1× PBS. The cell nuclei were stained with a Hoechst stain for 2 min at room temperature. The cells were washed 5 times in 1× PBS and maintained in 1× PBS for the duration of the imaging. As a control, the Jurkat cells were incubated for 24 h with 32 µM of BRACO-19, fixed, permeabilized, blocked, and stained with a secondary antibody as described above (in the absence of the primary anti-G4(1H6) antibody). The imaging was performed with the Leica TCS SP8 confocal microscope using the LAS X Life Science software (Leica Microsystems, Concord, Ontario, Canada). To quantify the G4 structures in the nucleus, a mask of each Hoechst-stained nuclei was generated and superimposed onto the fluorescent G4 channel of the same image using Fiji (ImageJ version 2.1.0/1.53c) [63]. The G4 fluorescence was measured for all of the nuclei in the field of view with an integrated intensity above 800. At least 430 cells from 40 fields of view per condition were measured from two independent experiments.

### 2.6. Flow Cytometry

Four days after infection with the HIV_GKO_ constructs, the Jurkat cells were stained with Zombie NIR^TM^ (BioLegend, #423105) to label the live cells and have them fixed with paraformaldehyde to a final concentration of 2%. The data were collected with a FACSCanto (Becton Dickinson, Franklin Lakes, NJ, USA) and the analyses were performed with FlowJo version 10.0.7 software (Flowjo, Ashland, OR, USA). The proportions of latent or productive integrations were calculated by dividing the percentage of the (GFP−,mKO2+) or (GFP +,mKO2+) cells (respectively) by the total percentage of positive cells ((GFP−,mKO2+)+(GFP+,mKO2+)). The percentage of (GFP−,mKO2−) or (GFP+,mKO2−) cells was not included when determining the proportion of latent and productive cells.

### 2.7. Datasets

Integration site datasets used in this study were independently analyzed using our in-house bioinformatics pipeline BLISIP v2.9. The Battivelli dataset was obtained from the “Integration Sites—Source Data” from reference [18]. The Achuthan dataset was obtained from the NCBI SRA using the accession number SRP132583, as described in [34]. All of the genomic sites in each dataset that hosted two or more sites (i.e., identical sites) were collapsed into one unique site for the analysis herein.

### 2.8. Statistical Analyses

All of the statistical tests were performed as described in the figure legends using Graphpad Prism 6 (Graphpad Software, San Diego, CA, USA).

## 3. Results

### 3.1. Reactivation Potential of HIV-1 Proviruses Correlates with Integration Site Placement near Non-B DNA Motifs

Recently, it was shown by Battivelli et al. (2018) that HIV-1 integration sites were distinguishable with respect to chromatin functional states and that these locations correlated with latency reactivation [18]. Given that several non-B DNA structures can influence chromatin organization and gene expression (e.g., G4, cruciform, triplex, and Z-DNA), we asked if latent provirus reactivation potential correlated with integration site placement near non-B DNA motifs. We defined ‘near’ as a window of the +/− 500 base pairs (bp) from the non-B DNA motif. The reason for selecting this distance was three-fold. First, we selected a distance with potential functional significance. For example, the region of the genome proximal to the TSS is essential for transcriptional regulation, and G4 motifs have been reported to be strongly enriched in the transcriptional regulatory region (defined as 500 bp upstream and downstream of the TSS) in warm-blooded animals [64,65]. Second, non-B DNA structures have been shown to influence positioning of the flanking nucleosomes encompassing 500 bp upstream and downstream of the structure, which equates to the length of DNA that wraps around approximately three nucleosomes on each side of the non-B DNA motif [66]. Third, a short distance was also selected to minimize the chance of multiple non-B DNA motifs being captured in the 500 bp window.

**Figure 1:**
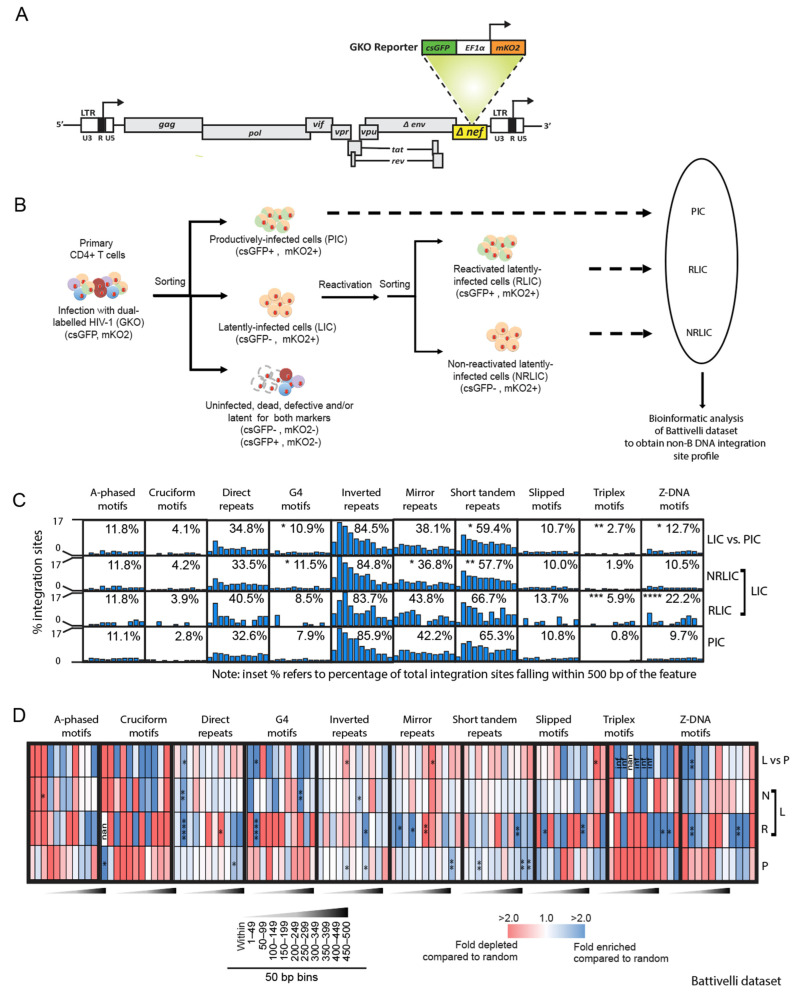
Integration near specific non-B DNA is associated with proviral reactivation in latently infected cells. (**A**), Schematic depicting the HIV-1 vector used to generate productively and latently infected cells. (**B**), Strategy used in the Battivelli study to isolate productively infected cells (PIC), latently infected cells (LIC), reactivatable latently infected cells (RLIC), and non-reactivatable latently infected cells (NRLIC) cell populations. (**C**), Graphs show the percentage of total unique HIV-1 integration sites located in or within 500 bp of various non-B DNA motifs (distributed in 50 bp bins). Inset numbers show the percentage of total unique integration sites falling within 500 bp of the non-B DNA motif. Statistical analysis is with respect to PIC. (**D**)**,** Heatmaps show the fold enrichment (blue) or depletion (red) of integration sites at each distance interval from the non-B DNA motif compared to the random control. The LIC population was compared to the PIC population. Fisher’s exact test; * *p* < 0.05, ** *p* < 0.01, *** *p* < 0.001, **** *p* < 0.0001. Infinite number (inf), 1 or more integrations were observed when 0 integrations were expected by chance. Nan, not a number (0 integrations were observed and 0 were expected by chance). PIC (P), RLIC (R), NRLIC (N), LIC (L), LIC versus PIC (L vs P).

The Battivelli integration site dataset was previously generated from primary CD4+ T cells infected with a dual-fluorescence HIV-1 reporter virus (HIV_GKO_) (Figure 1A) [18]. This virus was designed for the quantification and purification of a large number of latently infected cells by flow cytometry. Infection of the cells with HIV_GKO_ produced three populations of infected cells (Figure 1B). The first population contained productively infected cells (PIC) that expressed a codon-switched green fluorescent protein (GFP) under the control of the HIV-1 promoter in the 5′ long terminal repeat (LTR) and a distinct unrelated fluorescent protein mKO2 under the control of the EF1α promoter (GFP+, mKO2+) (Figure 1A,B). The second population contained latently infected cells (LIC) that express only mKO2 (GFP−, mKO2+). This LIC population was further divided into two additional sub-populations based on their αCD3/CD28 reactivation potential, cells that could be reactivated (RLIC), or cells that could not be reactivated (NRLIC) (Figure 1B). The third population of cells contained uninfected, dead, or defective cells (GFP+, mKO2−), and cells latent for both markers (GFP−, mKO2−). The third population of cells was excluded from further analyses.

We generated integration site profiles from the previously published Battivelli integration site dataset to determine if the reactivation potential of the HIV-1 proviruses correlated with integration site placement near non-B DNA motifs. For comparison, our integration site profile analysis was in agreement with the conclusions from the Battivelli study showing that the integration sites for the LIC population (NRLIC + RLIC) were enriched in regions of heterochromatin (e.g., satellite DNA and lamin-associated domains (LADs)) at a higher degree than the PIC population (Figure S1 and Tables S1 and S2). Analysis of our newly generated non-B DNA integration site profile showed that the majority of productive integration sites (PIC) were enriched near direct, inverted, mirror, and short tandem repeats (ranging from 33–86% of the total integration sites), followed by A-phased, slipped, G4, and Z-DNA motifs (8–11%), and a smaller percentage of sites within cruciform and triplex motifs (1–3%) (Figure 1C and Table S2). The LIC population exhibited a similar profile to that of the PIC population with the notable exception of reduced integration near the short tandem repeats (59% compared to 65%) and increased integration near the G4, triplex, and Z-DNA motifs (3–11% compared to 1–3%) (Figure 1C and Table S2).

Analysis of the RLIC and NRLIC populations separately showed that sites in the RLIC population were more enriched near triplex (6%) and Z-DNA (22%) motifs compared to the NRLIC population (2%, *p* < 0.05; and 11%, *p* < 0.001; respectively) (Figure 1C and Table S2). In addition, sites in the NRLIC population were enriched near the G4 motifs (12%) and reduced near mirror repeats (37%) and short tandem repeats (58%), compared to the RLIC population (9%, *p* > 0.05; 44%, *p* > 0.05; and 67%, *p* < 0.05; respectively).

To assess the distribution of the integration sites within the 500 bp window around the non-B DNA motifs, we sub-divided the 500 bp distance into 50 bp bins and quantified the integration sites in each bin (Figure 1C,D and Table S2). The distribution of sites in the PIC population were mostly uniformly distributed among the bins with an enrichment of sites in the bins that were more distal to direct, inverted, mirror, and short tandem repeats (~300–500 bp). In contrast, sites in the LIC population were enriched in bins more proximal (~1–100 bp) to direct repeats and G4, triplex, and Z-DNA motifs (Figure 1C,D and Table S2). Comparison of the RLIC and NRLIC populations separately showed that the RLIC population exhibited increased integration in the bins 1–50 bp away from direct repeats, mirror repeats, and G4, slipped, and Z-DNA motifs, and increased integration in the bins 350–500 bp away from the inverted repeats, short tandem repeats, triplex, and Z-DNA. Notably, the NRLIC population exhibited increased integration in the bin 350–399 bp away from G4 motifs compared to the RLIC population.

Together, these data show that different HIV-1 integration site biases exist towards non-B DNA motifs between productively and latently infected cells. Moreover, the reactivation of latent virus correlated with increased integration near mirror repeats, short tandem repeats, triplex, and Z-DNA motifs, and inversely correlated with integration near G4 motifs.

### 3.2. Pretreatment of Cells with G4 Ligands Alters HIV-1 Integration Targeting of G4 DNA

**Figure 2:**
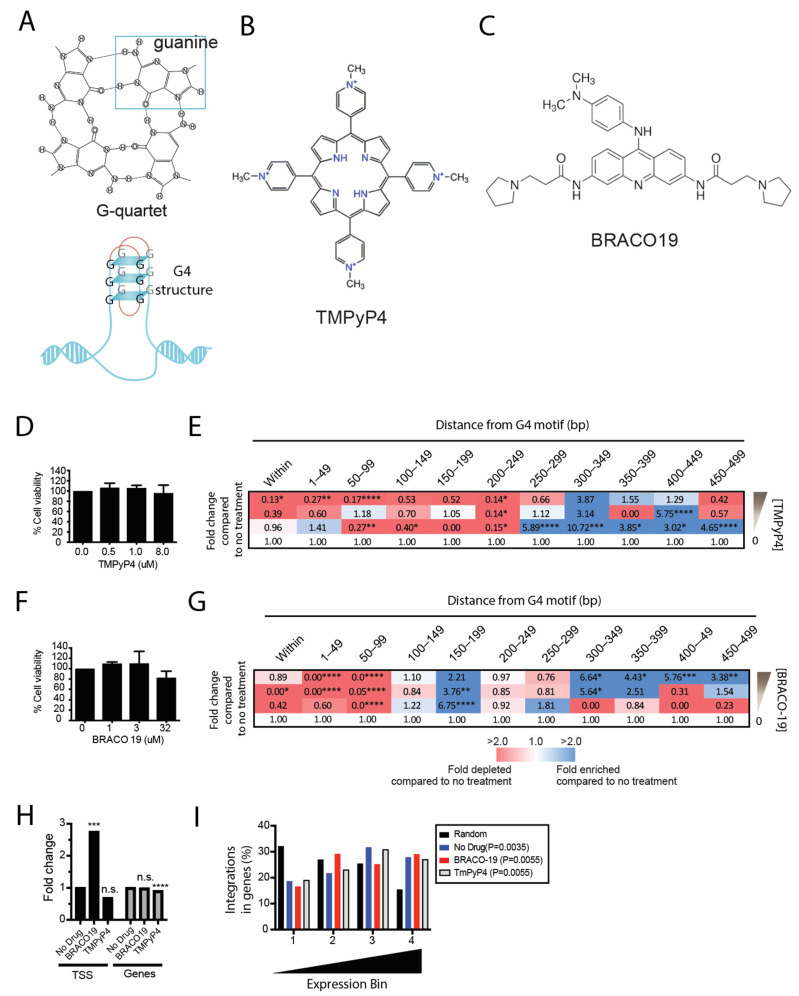
G4 structures influence integration site targeting in the genome. (**A**), Depiction of the nucleoside arrangement of a guanine-quartet and the G4 structure. (**B**), Structure of the TMPyP4 compound. (**C**), Structure of the BRACO-19 compound. (**D**), 293T cells were treated with increasing concentrations of TMPyP4 for 48 h and percent cell viability was determined using the MTT assay. Data are represented as mean ± SEM of at least 3 independent experiments. (**E**), Heatmap shows the fold enrichment (blue) or depletion (red) of integration sites at each 50 bp distance interval from the G4 motif compared to the untreated infected control cells. Statistical analysis is with respect to the untreated control. (**F**), 293T cells were treated with increasing concentrations of BRACO-19 for 48 h and percent cell viability determined using the MTT assay. Data are represented as mean ± SEM of at least 3 independent experiments. (**G**), Heatmap shows the fold enrichment (blue) or depletion (red) of integration sites at each 50 bp distance interval from the G4 motif compared to the untreated infected control cells. (**H**), Fold change in the percentage of integration sites located within 500 bp of transcription start sites (TSS) and directly in genes before and after treatment with BRACO-19 (32 µM) or TmPyP4 (8 µM). Statistical analysis is with respect to the untreated control. Fisher’s exact test; * *p* < 0.05, ** *p* < 0.01, *** *p* < 0.001, **** *p* < 0.0001. n.s., not significant. (**I**), Analysis of expression levels of genes targeted for integration with or without BRACO-19 (32 µM) or TmPyP4 (8 µM). Gene expression levels in 293T cells were obtained from the published GEO dataset GSE2451. A total of 22,288 genes were assayed and distributed into four equal bins by relative expression levels. The bin with the lowest average expression is at the left and the highest expression is at the right. Genes used as integration targets with or without treatment were distributed into their corresponding bins based on their expression levels and summed.

Given that the reagents to study G4 structures are more readily available than those for cruciform, triplex, and Z-DNA, we chose to further examine the influence of G4 DNA on HIV-1 integration site selection in vitro. G4 structures are four-stranded secondary structures containing four guanine bases that form a G-tetrad in a planar arrangement (Figure 2A) [40,67,68,69]. To determine the influence of G4 structures on integration site selection in the human genome during acute HIV-1 infection, we performed an experiment utilizing G4 structure-destabilizing and -stabilizing ligands TmPyP4 and BRACO-19, respectively (Figure 2B,C). The cationic porphyrin 5,10,15,20-tetra(N-methyl-4-pyridyl)porphin (TMPyP4) interacts with and destabilizes long-loop non-telomeric G4 structures while, paradoxically, stabilizing short-loop G4 structures located in the telomeric DNA [55,56,70,71,72,73]. We asked if the destabilization of non-telomeric G4 structures reduced integration near G4 DNA motifs. The control 293T cells or the cells treated with increasing concentrations of TMPyP4 were infected with HIV-1 pseudotyped with the vesicular stomatitis virus G envelope glycoprotein (HIV/VSV-G).

No significant reduction in the cell viability was detected (Figure 2D). Integration was significantly reduced within 500 bp of the G4 DNA motifs with increasing concentrations of TMPyP4 (Figure 2E and Tables S1 and S3). This reduced integration was observed despite an unexpected increase in the integration at low concentrations of TMPyP4 in a region 250–500 bp away from G4 DNA motifs.

BRACO-19 is a 3,6,9-trisubstituted acridine derivative that interacts with and stabilizes the G4 structures [54,72,74,75,76,77]. Although it was previously shown that BRACO-19 reduces but does not abolish reverse transcription at the template level due to the presence of a G4 motif at the 3′ end of the viral RNA genome [54], we asked if BRACO-19 treatment could alter integration targeting of the pre-integration complexes that are produced. Control 293T cells or cells treated with increasing concentrations of BRACO-19 were infected with HIV/VSV-G. No significant reduction in cell viability was detected after treatment with BRACO-19 (Figure 2F). Increasing concentrations of BRACO-19 resulted in a heterogeneous pattern of integration near the G4 motif where integration was significantly reduced within 100 bp and was significantly enriched 300–500 bp from the G4 DNA. Unexpectedly, integration was increased 150–199 bp from G4 DNA at low concentrations of BRACO-19, which then decreased at higher concentrations.

As a measure of specificity, integration in other commonly referenced genomic features showed that BRACO-19 treatment resulted in a 2.8-fold increase in integration sites near transcription start sites compared to the no drug control and TmPyP4-treated cells (Figure 2H). This finding was expected since TSS are rich in G4 DNA motifs [78,79,80,81,82]. No substantial change in the proportion of integrations sites within genes were observed with either treatment (Figure 2I). To determine if BRACO-19 or TmPyP4 treatment influenced the known preference of HIV-1 for integration into highly expressed genes, we analyzed a previously published gene expression profiling dataset for 293T cells (GSE2451). The genes were categorized into four different expression bins ranging from a low- to high-level of gene expression. Integration sites from the cells exhibited a significant enrichment in highly expressed genes regardless of the treatment. The analysis of the integration sites along each chromosome in the presence or absence of either treatment revealed no integration sites located in the telomeric regions, and thus did not appear to alter the normally low level of HIV-1 integration in telomeres (Figure S2 and Table S5). However, since the integration sites that could not be unambiguously mapped to a single region in the genome were excluded from analysis, it is possible that the sites in the telomeric repeats were present but undetected.

Together, these data show that pre-treatment of cells with G4 structure-stabilizing or -destabilizing ligands reduced integration within 100 bp of the motifs, whereas the stabilizing ligand BRACO-19 increased integration 300–500 bp away from the motif.

### 3.3. HIV-1 Favors G4 Structures with Long-Loops for Integration

**Figure 3:**
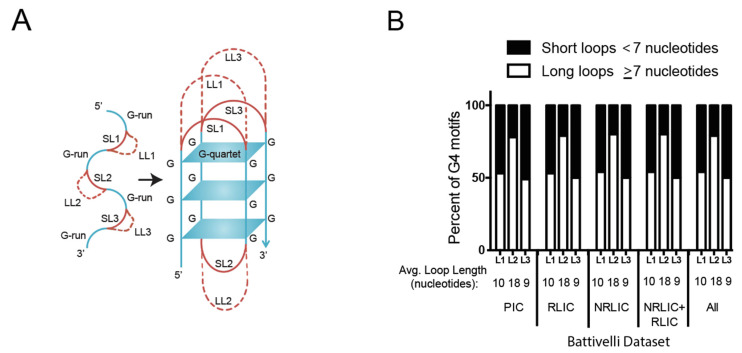
Integration in or near G4 motifs favors G4 structures with long-loops. (**A**), Schematic depicting a G4 motif (left) and G4 structure (right) consisting of four adjacent runs of two or more guanines, with three loop regions of nucleotide subsequences (L1, L2, and L3) connecting the G-runs. Loops containing <7 nucleotides are considered short-loop (SL) G4s (solid red line), whereas ≥7 are considered long-loop (LL) G4s (dashed red line). (**B**), G4 motif sequences located within 500 bp of an integration site were isolated from the Battivelli datasets and the loop-lengths were calculated. The percentage of G4 motifs in each dataset classified as short-loops or long-loops were compared in the bar graphs. The average loop lengths for each of the three loops were calculated and are shown below each bar graph.

Putative G4 structures are identified using the motif G_x_N_y1_G_x_N_y2_G_x_N_y3_G_x_ [83,84,85,86]. The motif consists of four guanine tracts with three intervening loops (Figure 3A). In this expression, x represents the number of guanine nucleotides, Ny1–Ny3 represent the three intervening loops and can be categorized as short-loop G4 structures (1–7 nucleotides), or long-loop G4 structures (≥7 nucleotides) based on the number of nucleotides (N) in the loop. The typical loop lengths in the human genome are between 1 and 7 nucleotides [87]. The loop-length has been shown to play an important role in the G4 structure’s stability and protein-binding specificity [85,88]. We asked if the HIV-1 integration sites observed in the PIC, LIC, RLIC, and NRLIC populations from the Battivelli study were biased towards short- or long-loop G4 DNA motifs and if loop-length correlated with reactivation potential of latently infected cells. The G4 DNA sequences were extracted from the Battivelli dataset and the average loop-lengths were compared for each of the three loops. No difference in the loop-lengths was observed between the PIC, RLIC, or NRLIC populations (Figure 3B). Notably, loop two was twice as long as loops one and three within each population of cells. These data indicate that HIV-1 integration is biased towards long-loop G4 motifs and that the loop length does not correlate with productive or latent integration, or the reactivation potential of latently infected cells.

### 3.4. Stabilization of G4 Structures In Vitro Increases the Proportion of Latently Infected Cells

**Figure 4:**
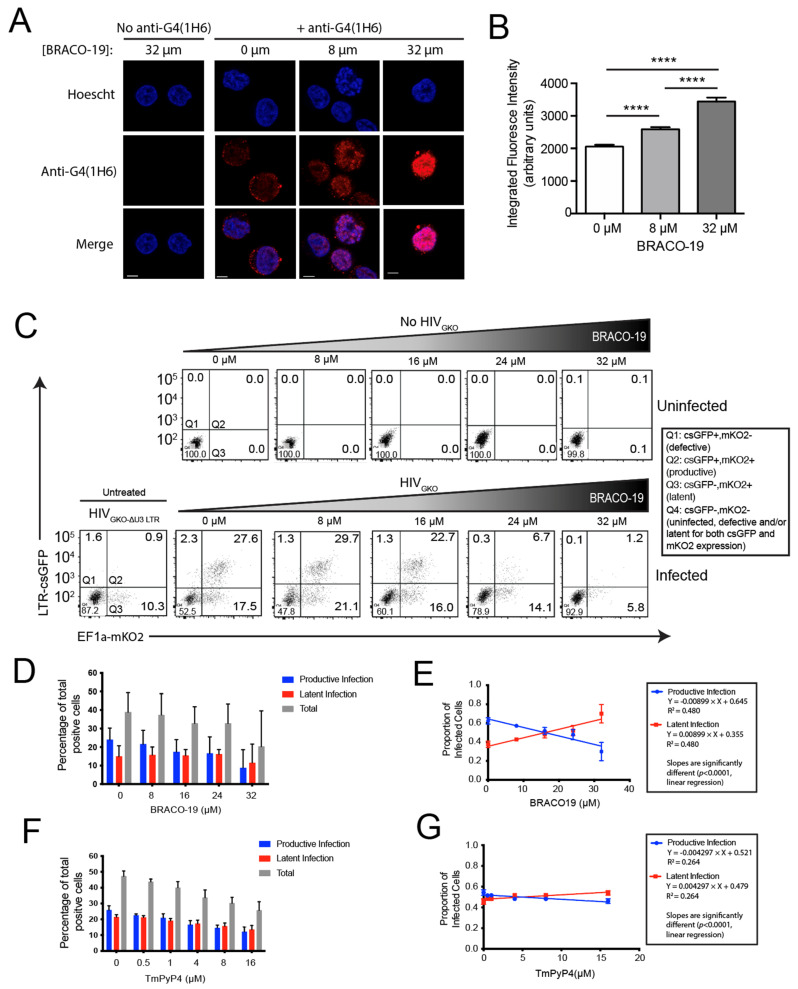
Stabilization of G4 structures increases the proportion of latently infected cells in vitro. (**A**), Jurkat cells were pre-treated for 24 h with increasing concentrations of BRACO-19 and then subjected to confocal immunofluorescence microscopy using anti-G4(1H6) antibody. Nuclei were identified with Hoechst staining. (**B**), Nuclear G4 structure content was quantified by creating a mask of Hoechst nuclear staining for all nuclei using FIJI (NIH). Each mask was overlaid onto the G4 staining channel of the same image and the G4 staining was measured for each nucleus in the field of view with an integrated intensity above 800. Data shown represent the average (+/− S.E.M.) of at least 430 cells per condition from 40 fields of view from two independent experiments. One-way ANOVA with Tukey’s multiple comparisons test; ****, *p* < 0.0001. (**C**), Jurkat cells were pre-treated for 24 h with increasing concentrations of BRACO-19 and infected with HIV_GKO_ or HIV_GKO-DU3LTR_ for 96 h in the presence of drug. Cells were stimulated with anti-CD3/CD28. Live cells were gated on using Zombie NIR^TM^ staining and the percentage of total fluorescence positive cells (**D**), and the proportion of productively infected (GFP+, mKO2+) and latently infected (GFP−, mKO2+) cells (**E**), were determined using flow cytometry. (**F**,**G**), Jurkat cells were similarly pre-treated for 24 h with increasing concentrations of TmPyP4 and infected with HIV_GKO_ or HIV_GKO-DU3LTR_ for 96 h in the presence of drug. The percentage of total fluorescence positive cells (**F**) and the proportion of productively infected (GFP+, mKO2+) and latently infected (GFP−, mKO2+) cells (**G**) were determined using flow cytometry. Data shown represent the average (+/− S.E.M.) from 3 independent experiments. Statistical analysis was performed using linear regression.

G4, cruciform, triplex, and Z-DNA have been shown to silence the expression of adjacent genes [42,43,44,45,46,47,48,49,50,51,52]. Given our finding that integration sites are enriched near G4 DNA motifs in latently infected cells and that altering the G4 DNA stability influences integration targeting of G4 DNA motifs, we asked if the stabilization of G4 structures in vitro increases the proportion of the latently infected cells in a pool of infected cells. Twenty-four hours prior to the infection, we treated the Jurkat cells with increasing concentrations of BRACO-19 to stabilize the intracellular G4 structures. The stabilization of nuclear G4 structures was verified by confocal immunofluorescence microscopy using anti-G4(1H6), which binds specifically to the G4 DNA structures [62,89]. As expected, nuclear G4 staining increased with increasing concentrations of BRACO-19 (Figure 4A,B). Some G4 DNA staining was also observed in the cytoplasm, which is consistent with previous observations and is likely attributed to the release of G4 DNA structures into the cytoplasm after DNA damage repair processes [90]. No auto-fluorescence was observed with BRACO-19 at the highest concentration used (32 µM) (Figure 4A).

To measure the number of productively and latently infected cells in a pool of infected cells, we infected Jurkat cells with the HIV_GKO_ dual-reporter virus (VSV-G pseudotyped), as described above (see Figure 1A,B) [18]. Productively infected cells were double-positive for GFP and mKO2 fluorescence (GFP+, mKO2+), whereas latently infected cells were positive for mKO2 fluorescence only (GFP−, mKO2+). Cells exhibiting only GFP expression (GFP+, mKO2−) were considered defective. Cells not expressing either marker (GFP−, mKO2−) comprise uninfected or dead cells, cells containing defective proviruses, and/or cells containing proviruses latent for both a GFP and mKO2 expression. As a control, we utilized the same HIV_GKO_ virus lacking the U3 promoter region of the 3′ LTR (HIV_GKO-DU3LTR_), which results in an integrated virus devoid of the 5′ HIV U3 region (GFP−, mKO2+).

To test the effect of stabilizing G4 structures on the proportion of productively and latently infected cells, the Jurkat cells were treated with BRACO-19 for 24 h and then infected with HIV_GKO_ for 4 days. The live cells were identified by flow cytometry using Zombie NIR^TM^ staining and analyzed for GFP and mKO2 fluorescence. As expected, infection with the HIV_GKO-DU3LTR_ control virus yielded >99% (GFP−, mKO2+) cells (Figure 4C). Additionally, as expected, increasing concentrations of BRACO-19 caused a reduction in, but not an abolishment of, infection due to its inhibitory activity on reverse transcription (Figure 4C,D). Analysis of the infected cells (mKO2+) showed that the proportion of productively infected cells (GFP+, mKO2+) decreased from 60% to 22%, whereas the proportion of latently infected cells (GFP−, mKO2+) increased from 40% to 78% (*p* < 0.0001, linear regression; *R*^2^ = 0.480, DFn = 1, DFd = 44, F = 40.57,) (Figure 4D,E). In comparison, analysis of the infected cells treated with increasing concentrations of TmPyP4 showed a more modest effect compared to the BRACO-19 treatment, where the proportion of the productively infected cells (GFP+, mKO2+) decreased from 54% to 46%, and the proportion of the latently infected cells (GFP−, mKO2+) increased from 46% to 54% (*p* < 0.0001, linear regression; *R*^2^ = 0.264, DFn = 1, DFd = 56, F = 20.06) (Figure 4F,G). This finding suggests that G4 DNA is not the only factor contributing to latent infection. Similar to BRACO-19, TmPyP4 also caused a reduction in, but not an abolishment of, infection (Figure 4F). The mechanism(s) underlying this anti-HIV activity of TmPyP4 has not been fully characterized. TmPyP4 may interfere with important interactions between a G4 structure located in the HIV DNA flap region and nucleocapsid protein during nucleocapsid assembly, and/or it may interfere with the interstrand quadruplex formation in the dimerization of HIV RNA [91,92].

Together, these data show that the stabilization of nuclear G4 structures with BRACO-19 increases the number of latently infected cells and decreases the number of productively infected cells in a population of HIV-1 infected cells in vitro.

### 3.5. CPSF6 and LEDGF/p75 Influence Integration near Non-B DNA Motifs

**Figure 5:**
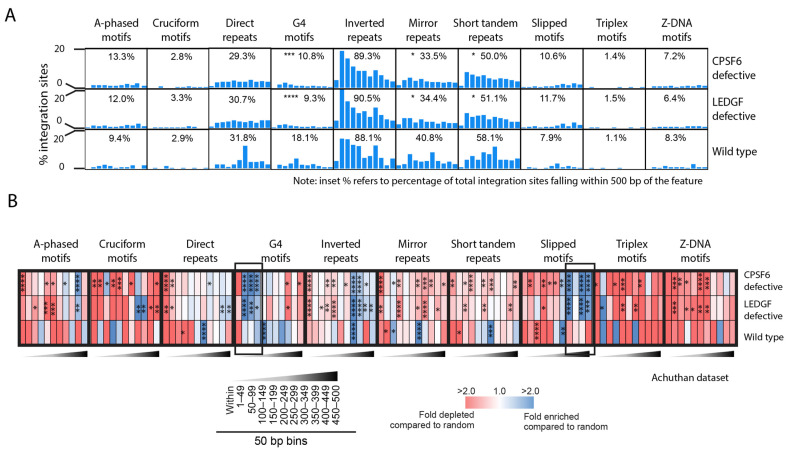
CPSF6 and LEDGF/p75 promote integration near several non-B DNA. (**A**), Graphs show the percentage of total unique HIV-1 integration sites located in or within 500 bp of various non-B DNA motifs (distributed in 50 bp bins) from wild type (control), CPSF6 depleted, or LEDGF/p75 depleted cells. Inset numbers show the percentage of total unique integration sites falling within 500 bp of the non-B DNA motif. Statistical analysis is with respect to wild type. (**B**), Heatmap shows the fold enrichment (blue) or depletion (red) of integration sites at each distance interval from the non-B DNA motif compared to the random control. Black boxes highlight notable regions of enriched integration. Statistical analysis is with respect to the random control. Fisher’s exact test; * *p* < 0.05, ** *p* < 0.01, *** *p* < 0.001, **** *p* < 0.0001.

Since LEDGF/p75 and CPSF6 promote integration into actively transcribed genes residing in gene-dense regions, we asked if CPSF6 and LEDGF/p75 influence the targeting of non-B DNA for integration [27,34,58,93,94,95]. We analyzed a previously well-characterized integration site dataset by Achuthan et al. (2018) who studied the impact of CPSF6 and LEDGF/p75 on integration site targeting [34]. In that study, CPSF6 function was inhibited by using the HIV-1 capsid mutant A77V, which impairs CPSF6 binding efficiency without severely decreasing infectivity. The LEDGF/p75 function was inhibited by treating the cells at the time of the infection with the allosteric integrase inhibitor BI-D, which competes with integrase-LEDGF/p75 binding. We generated the non-B DNA integration site profiles using this independently generated previously published dataset and asked if CPSF6 and/or LEDGF/p75 potentially influenced integration site targeting of non-B DNA motifs. Indeed, the cells defective for CPSF6 or LEDGF/p75 function exhibited significantly reduced integration within 500 bp of G4 DNA, mirror repeats, and short-tandem repeats compared to the wild type controls (Figure 5A, Tables S1 and S4). Analysis of the distribution of sites near non-B DNA features revealed an unexpected enrichment of integration sites 1–150 bp from G4 DNA in cells defective for the CPSF6 or LEDGF/p75 function compared to wild type cells (Figure 5A,B and Table S4). In addition, integration was more enriched 300–500 bp from slipped DNA in cells defective for CPSF6 and LEDGF/p75 function compared to wild type cells. Together, these data suggest that functional CPSF6 and LEDGF/p75 influence the distribution of the integration sites near several non-B DNA features.

## 4. Discussion

The ability of HIV-1 to target non-B DNA structures for integration has several important implications for productive and latent infection, especially since genomic position effects have been shown to influence HIV-1 expression and latency reversal [18,20,30]. Several non-B DNA structures are associated with active genes in vivo and contribute to a dynamic interplay between DNA structure, chromatin organization, and transcriptional activities [67]. Non-B DNA structures can be recognized by non-B DNA-specific transcription factors, leading to transcriptional activation [96,97,98,99]. Conversely, the unusual non-B DNA structure can also block the binding of B-DNA-specific transcription factors, resulting in constitutive repression of adjacent genes and proviruses [43,50,51,89,100,101,102]. As such, the proximity of integrated proviruses to non-B DNA structures is likely to influence proviral expression, including reactivation potential by the LRAs. Indeed, we identified different integration site biases toward non-B DNA between latent and productive proviruses, especially those known to influence gene transcription (e.g., G4 DNA, triplex DNA, and Z-DNA).

The factors influencing the reactivation potential of latent proviruses are not fully understood. Latent proviruses that have a stronger response to LRAs tend to be located closer to active regulatory elements such as histone H3 acetylated at K27, monomethylated at K4, or trimethylated at K4 or K36, whereas non-reactivatable proviruses are biased toward heterochromatin with histone H3 trimethylated at K9 or K27 [18,20]. Non-B DNA structures, particularly G4 structures, have been shown to fine-tune chromatin organization within cells, although the mechanism underlying this activity is not fully understood [67]. Non-B DNA sequences are thought to alter the intrinsic sequence preference of nucleosomes, thereby affecting nucleosome occupancy [67,103]. Similarly, non-B DNA structures might sterically exclude nucleosomes, thereby affecting nucleosome positioning [67,104]. Indeed, G4 DNA has been shown to form in nucleosome-free regions in the genome [105]. Future research will help determine if the ability of G4 structures to locally and dynamically organize flanking nucleosomes also impacts the chromatin functional state near integrated HIV-1 proviruses, thereby influencing the transcriptional activity of those adjacent proviruses.

Other position effects such as the proximity of the integrated proviruses to enhancers can also impact proviral expression. Chen et al. (2017) showed that HIV-1 proviral expression is strongest when the proviruses are inserted close to endogenous enhancers, whereas latent insertions are mapped further away from enhancers [20]. We identified a strong correlation between latent proviruses that could be reactivated with LRAs and their integration within 50 bp of specific non-B DNA features that influence gene expression (e.g., G4 and Z-DNA). Indeed, enhancer–promoter interactions have been shown to be facilitated by G4 DNA [106]. It will be interesting to learn if G4 DNA located +/− 500 bp from latent proviral integration sites facilitates interactions with enhancers to render these proviruses more responsive to LRAs.

It has been previously suggested that an optimally tuned bias for integrating into transcriptionally active (euchromatin) versus inactive (heterochromatin) regions of the genome may help establish a diverse latent viral reservoir [107,108,109]. CPSF6 and LEDGF/p75 are two host proteins that promote integration into euchromatin [32,34,110,111,112]. Work performed by Vranckx et al. (2016) showed that by blocking LEDGF/p75–integrase interaction with the drug LEDGIN, integration site preferences shifted away from actively transcribed genes near the nuclear pores to the inner nuclear compartment [30]. Integration into transcriptionally silent heterochromatin is often located close to the nucleolus in the inner core and LADs at the nuclear periphery [111]. It is widely known that heterochromatin is tightly wrapped by distinctive DNA-binding proteins (e.g., histones) and that this chromatin environment is generally unfavorable for integration. Interestingly, G4 structures are found in heterochromatin but are typically not located within the DNA wrapped around a histone octamer [89,105,113]. It is possible that this property creates a partially open state in the regions of heterochromatin, allowing an integration to occur near these structures.

Although the targeting of non-B DNA by pre-integration complexes likely involves multiple factors given the diversity of the non-B DNA, we identified LEDGF/p75 and CPSF6 from previously published integration site profiles as potential candidates that alter the targeting of some non-B DNA motifs. It could be that LEDGF/p75 and CPSF6 simply promote interactions between the pre-integration complexes and euchromatin where it then integrates near non-B DNA via another unknown mechanism mediated by, for example, certain non-B DNA binding proteins. Another possibility is that LEDGF/p75 and CPSF6 themselves can recognize specific non-B DNA structures to promote interactions between the pre-integration complex and the genomic DNA, leading to integration. More research is needed to investigate these and other possibilities.

The distribution of integration sites near non-B DNA was often heterogeneous and not located at precise nucleotide distances from the features. One explanation could be that the pre-integration complex is first attracted to the non-B DNA structure where it then integrates non-discriminately near the feature. Other explanations for the heterogeneity could be differences in the cellular transcriptional profiles that could affect the formation of non-B DNA structures, the adjacent nucleosome occupancy, and/or the DNA-binding proteins that generate steric constraints for an integration. Moreover, genetic polymorphisms (e.g., insertions/deletions) near non-B DNA motifs may also contribute to differences in the distance of the integration sites to the features in infected individuals. Interestingly, when we treated cells with low concentrations of G4 DNA stabilizing or destabilizing ligands, we observed increased integration in the windows of 150–200 bp (BRACO-19) and 250–500 bp (TmPyP4) around the motifs. It is intriguing that these distances correlate with the approximate distance of one, two, or three nucleosomes away from the G4 motif. The reason for this increase is currently unknown. One possible explanation is that at these low concentrations, the binding of the ligands perturbs the G4 structures and disrupts the G4 DNA- and/or nucleosome-binding proteins that typically block integration in these regions. Another possibility is that perturbation of these G4 DNA structures at these low concentrations leads to the temporary formation of alternative non-B DNA structures that promote integration. For example, it was shown by Masai et al. (2018) that some G4 DNA motifs may generate G4 structures that undergo a dynamic transition from one structure to another, with the potential to generate multiple G4-like structures on one or both DNA strands [114].

Previous searches to identify a consensus sequence for integration site targeting have only revealed an apparent weak palindromic sequence at the site of the insertion of several retroviruses [115,116,117,118,119,120,121]. Recent work by Kirk et al. (2016) challenged this notion by showing that the palindromic consensus sequence arises in the population average as a consequence of the non-palindromic motifs existing in equal proportions on the plus and minus strand of the target sequence [122]. Our study not only supports the notion that there is not likely a single palindromic consensus sequence at the integration site but shows that the integration site target sequences are heterogeneous in nature, many of which fall near non-B DNA structure-forming motifs. The inspection of each of the non-palindromic sequences identified by Kirk et al. (2016) in the subpopulations of the target integration sites from HTLV-1, HIV-1, MLV, ASLV, and PFV(IV) revealed that they all represent different non-B DNA sequences that are predicted to form slipped DNA structures. Moreover, the HTLV-1 ‘weak palindromic’ consensus site sequence in Kirk et al.’s study that predominates from the population average is in fact a non-B DNA slipped motif [115,116,120,122]. This suggests that despite having distinct nucleotide consensus sequences (palindromic or non-palindromic), the nature of the sequence is predicted to form a non-B DNA structure. Our findings using G4-stabilizing and -destabilizing ligands further highlights the likelihood that it may not be the primary DNA sequence itself that plays an important role in attracting the HIV-1 pre-integration complex, but rather it is the secondary structure formed by the non-B DNA motif itself that is important. Short-loop G4 motifs (e.g., (TTAGGG)_n_) are highly enriched in the telomeres of the chromosomes, in which no strong bias for a HIV-1 integration has been observed. Our finding that HIV-1 demonstrated an integration bias for the long-loop G4 structures as opposed to the short-loop G4 structures raises the possibility that structural variations in some of these non-B DNA structures may also influence integration.

## 5. Conclusions

We have shown that different integration site biases for non-B DNA exist between productive and latent HIV-1 infection and correlate with the reactivation potential of latent proviruses. The manipulation of G4 DNA also altered the proportion of productively and latently infected cells in vitro. Moreover, we identified two key proteins involved in the integration process, LEDGF/p75 and CPSF6, as potential candidates that influence the distribution of integration sites near several non-B DNA motifs. Targeting non-B DNA structures directly as a form of antiretroviral therapy would be difficult given the abundance of non-B DNA in the human genome. However, a better understanding of how pre-integration complexes interact with non-B DNA may identify viral components critical for the selection of non-B DNA. These components could be targeted with novel small-molecule inhibitors to alter the proportion of productive and latent infections during an acute infection, and/or to enhance ‘shock and kill’ and ‘block and lock’ therapies in the future.

## Data Availability

The sequences reported in this paper will be deposited in the National Center for Biotechnology Information Sequence Read Archive (NCBI SRA) (SRP164286: SRR7975450-SRR7975468).

## Supplementary Materials

The following supporting information can be downloaded at: https://www.mdpi.com/article/10.3390/v14112494/s1, Figure S1: Integration site profile for NRLIC, RLIC, and PIC populations; Figure S2: Circa plot showing the distribution of integration sites on each chromosome (hg19).Table S1: List of integration site datasets used in this study; Table S2: Integration site distribution from productively infected cells (PIC), reactivated latently infected cells (RLIC), and non-reactivated latently infected cells (NRLIC) from the Battivelli dataset; Table S3: Integration site distribution with respect to G4 motifs in cells treated with increasing concentrations of G4-stabilizing and -destabilizing ligands; Table S4: Integration site distribution from HIV-1 infected wild type (WT), LEDGF defective (BID), and CPSF6 defective (A77V) cells from the Achuthan dataset; Table S5: List of integration sites located in telomeres.
