# Supplementary material for: G-Quadruplex DNA and Other Non-Canonical B-Form DNA Motifs Influence Productive and Latent HIV-1 Integration and Reactivation Potential
Source: Viruses. 2022 Nov 11;14(11):2494. doi: 10.3390/v14112494 (PMC9692945; doi:10.3390/v14112494)
Supplement: Supplementary file 1 [file viruses-14-02494-s001.zip › Supplemental Figs and Table1.pdf]

SUPPLEMENTAL INFORMATION:

Supplemental Figure Legends:

Supplemental Figure 1: Integration site profiles from productively infected cells (PIC), reactivated latently infected cells (RLIC), and non-reactivated latently infected cells (NRLIC) from the Battivelli dataset. Heatmap depicting the fold enrichment or depletion of integration sites in common genomic features compared to the MRC. The ‘RLIC+NRLIC’ population was compared to the PIC population. * $P < 0.05$, ** $P < 0.01$, *** $P < 0.001$, **** $P < 0.0001$. Infinite number (inf), 1 or more integrations were observed when 0 integrations were expected by chance. Not a number (nan), 0 integrations were observed and 0 were expected by chance. Related to Figure 3.

Figure S1

PIC

	Within	1-499	500-4999	5000-49999	>50000
CpG Islands	0.48	0.89	2.43****	1.82****	0.59****
DNaseI hypersensitivity sites	1.35****	1.37****	0.77****	0.14****	0.04****
Endogenous retroviruses	0.62***	0.68***	0.78****	1.97****	1.16
Long interspersed nuclear elements	0.84*	0.96	1.16****	1.96****	0.00**
Low complexity repeats	0.83	0.69**	1.15****	1.10*	0.11**
RefSeq genes	1.90****	0.54	0.96	0.44****	0.23****
Satellite DNA	0.39	4.44*	1.39	1.50*	1.20*
Simple repeats	1.80**	1.19*	1.06*	0.85	0.00**
Short interspersed nuclear elements	1.5****	1.5****	0.74****	0.23****	0.14*
Transcription start sites	0.00	1.20	2.19****	1.54****	0.42****
UCSC genes	1.89****	0.27*	0.78	0.46****	0.27****

RLIC

CpG Islands	0.00	3.75	1.76*	1.56****	0.76*
DNaseI hypersensitivity sites	1.64**	1.36**	0.68**	0.17*	0.24
Endogenous retroviruses	0.72	1.09	0.88	1.55***	0.59
Long interspersed nuclear elements	0.94	0.98	1.07	2.42**	0.00
Low complexity repeats	1.67	1.03	0.92	1.30*	0.59
RefSeq genes	1.74****	0.00	0.88	0.47**	0.58**
Satellite DNA	2.50	0.00	0.00	1.52	1.61*
Simple repeats	2.22	1.44*	0.94	0.99	0.00
Short interspersed nuclear elements	1.67**	1.43**	0.69***	1.08	0.77
Transcription start sites	nan	1.00	2.43****	1.23*	0.65***
UCSC genes	1.69****	0.00	1.06	0.49**	0.56**

NRLIC

CpG Islands	0.60	1.33	1.61***	1.44****	0.86**
DNaseI hypersensitivity sites	1.17	1.34****	0.79****	0.36***	0.78
Endogenous retroviruses	0.98	1.16	0.95	1.30****	0.92
Long interspersed nuclear elements	0.59****	1.15*	1.26****	1.25	0.16*
Low complexity repeats	0.71	0.91	1.07	1.11	1.20
RefSeq genes	1.39****	1.76	0.77	0.87	0.77***
Satellite DNA	4.48****	2.50	0.69	1.25	0.93
Simple repeats	2.39****	1.07	1.06	0.96	0.00**
Short interspersed nuclear elements	1.42***	1.15*	0.93	0.80	0.92
Transcription start sites	0.00	0.80	1.68****	1.20***	0.85**
UCSC genes	1.38****	1.39	0.72	0.90	0.78**

(NRLIC+RLIC) vs. PIC

CpG Islands	1.16	1.16	0.73*	0.78****	1.45****
DNaseI hypersensitivity sites	0.91	0.99	1.03	2.31	8.09*
Endogenous retroviruses	1.56*	1.60***	1.23***	0.66****	1.04
Long interspersed nuclear elements	0.78*	1.15	1.05	0.76	inf
Low complexity repeats	1.16	1.39*	0.93	0.99	12.71**
RefSeq genes	0.75****	1.73	0.81	1.75****	3.29****
Satellite DNA	8.09**	0.29	0.46	0.86	0.87
Simple repeats	1.22	1.03	0.96	1.13	nan
Short interspersed nuclear elements	0.95	0.85**	1.14*	3.47***	9.25*
Transcription start sites	nan	0.58	0.82*	0.77****	1.98****
UCSC genes	0.75****	2.89	0.99	1.69***	2.91****

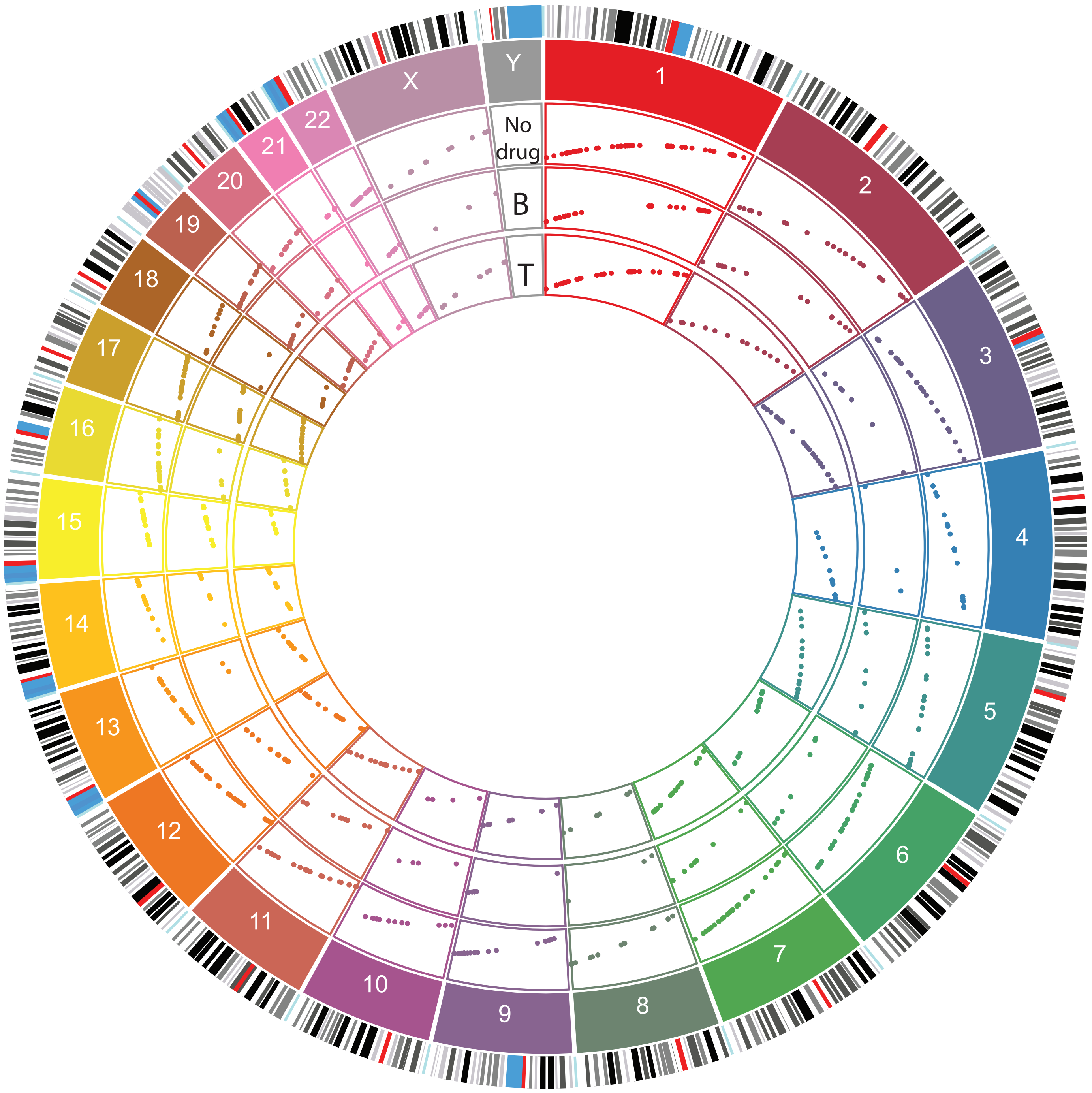

Figure S2: Circa plot showing the distribution of integration sites on each chromosome (hg19). The rings (from outer to inner) depict the cytoband pattern, chromosome numbers, location of integration sites in the absence of treatment ('No Drug'), location of integration sites in the presence of BRACO-19 (32 uM) ('B') and location of integration sites in the presence of TmPyP4 (8 uM) ('T').

Table S1: List of integration site datasets used in this study.

Dataset	Group	# of unique integration sites	Cell Type	Reference
Battivelli	Productively Infected (PIC)	950	Primary CD4+ T cells	Battivelli et al. (2018)
	Reactivated Latently Infected (RLIC)	153	Primary CD4+ T cells	
	Non-Reactivated Latently Infected (NRLIC)	669	Primary CD4+ T cells	
Achuthan	Wild type (WT)	277	293T cells	Achuthan et al. (2018)
	LEDGF/p75 depletion (BID)	2949	293T cells	
	CPSF6 depletion (A77V)	4431	293T cells	
This study	Untreated	2017	293T cells	This study
	BRACO19 (1 uM)	797	293T cells	
	BRACO19 (3 uM)	1073	293T cells	
	BRACO19 (32 uM)	759	293T cells	
	TMPyP4 (0.5 uM)	1223	293T cells	
	TMPyP4 (1 uM)	1286	293T cells	
	TMPyP4 (8 uM)	1302	293T cells	
